# Personal Activities, Social Participation and Functional Disability in Aged Living Alone with Fall Experience

**DOI:** 10.1093/geroni/igaa057.3375

**Published:** 2020-12-16

**Authors:** SuJung Jung, Sunghee Tak

**Affiliations:** Seoul National University, Seoul, Republic of Korea

## Abstract

Functional disability leads to limitations in the older adults’ personal activities and social participation. The purpose of this study was to examine the International Classification of Functioning Disability and Health(so called ICF) model in which personal activities and social participation influence functional disability in older adults who live alone and have experienced falls. The study used a secondary data analysis of the 2017 National Survey of Older Koreans. A total of 501 study participants met the inclusion criteria. The results of multiple linear regression indicated that gender and the number of acquaintances were significantly related to the functional disability of social participation while overnutrition, depressed symptom and cognitive dysfunction were related to the functional disability of personal activities. Lastly, poor muscle strength, old age and economic status were predictors of the functional disabilities of both personal activities and social participation. The findings of the study revealed that it is important to comprehensively evaluate not only personal activities but also social participation of older adults who live alone and have experienced falls. In addition, the ICF model may be useful in the development of intervention programs for preventing functional disability in the population.

